# Development of a loop-mediated isothermal amplification assay for the rapid detection of *Russula subnigricans* and *Russula japonica*

**DOI:** 10.3389/fmicb.2022.918651

**Published:** 2022-08-23

**Authors:** Pan Long, Zijuan Jiang, Zhengmi He, Zuohong Chen

**Affiliations:** College of Life Science, Hunan Normal University, Changsha, China

**Keywords:** loop-mediated isothermal amplification, *Russula subnigricans*, *Russula japonica*, ITS, mushroom poisoning

## Abstract

*Russula subnigricans* is the only deadly species in the genus *Russula* with a mortality rate of more than 50%, and *Russula japonica* is the most common poisonous species, making rapid species identification in mushroom poisoning incidents extremely important. The main objective of this study was to develop a rapid, specific, sensitive, and simple loop-mediated isothermal amplification (LAMP) assay for the detection of *R. subnigricans* and *R. japonica*. Two sets of species-specific LAMP primers targeting internal transcribed spacer (ITS) regions were designed to identify *R. subnigricans* and *R. japonica*. The results demonstrated that while LAMP could specifically detect *R. subnigricans* and *R. japonica*, the polymerase chain reaction (PCR) could not distinguish *R. subnigricans* from *Russula nigricans*. In addition, the results demonstrated that, compared to electrophoresis-LAMP and real-time quantitative LAMP (RT-qLAMP), the detection sensitivity of HNB-LAMP (a mixture of LAMP with hydroxy naphthol blue (HNB) dye) for *R. subnigricans* could reach 0.5 pg/μl and was 100-fold higher than that of PCR. The LAMP reaction could be completed in 45 min, which is much faster than the conventional PCR. In the future, LAMP can be used a quick, specific, and sensitive detection tool in various fields.

## Introduction

Mushroom poisoning is a public health emergency in China, with the highest mortality rate and the highest number of food poisoning deaths. To date, 1,662 taxa of fungi have been checked and corrected in China, including 1,020 edible fungal taxa, 692 medicinal fungal taxa, and 480 toxic fungal taxa (Wu et al., 2019). Between 2004 and 2014, there were 576 mushroom poisoning cases in mainland China, with 3,701 patients and 786 deaths (Zhou et al., 2016). Between 2019 and 2021, the Chinese Center for Disease Control and Prevention reported 1,279 cases of mushroom poisoning, with 3,411 patients and 67 deaths (Li et al., 2020, 2021, 2022).

*Russula* Pers., one of the largest genera of ectomycorrhizal fungi, contains both edible and poisonous species (Buyck et al., 2018). According to records, the edible taxa of the *Russula* genus are typically *Russula virescens, Russula griseocarnosa, Russula delica*, and others, and 18 poisonous taxa have been reported (Wu et al., 2019; Wang, 2020). In China, the most common cause of mushroom poisoning is rhabdomyolysis caused by accidental eating of *Russula subnigricans* as well as the fatal deaths caused by the lethal amanitas, which causes acute liver damage, or species in the genera *Lepiota* and *Galerina*. Between 1994 and 2012, Chen et al. (2014) investigated 102 cases of mushroom poisoning in southern China and found that *R. subnigricans* is one of the three mushrooms responsible for the majority of deaths, with a mortality rate of more than 50%. *R. subnigricans* is the only lethal mushroom of the *Russula* genus that causes rhabdomyolysis, and it has been linked to numerous poisoning deaths in the last 20 years (Lee et al., 2001; Matsuura et al., 2009; Cho and Han, 2016; Li et al., 2020, 2021, 2022). The most common species in China is *Russula japonica*, which causes gastroenteritis when consumed. According to the reports published by the China CDC between 2019 and 2021, the top three species that cause clinical syndromes associated with gastroenteritis are *Chlorophyllum molybdites, R. japonica*, and *Entoloma omiense*, with *R. japonica* ranking second for three consecutive years (Li et al., 2020, 2021, 2022).

**Figure 1:**
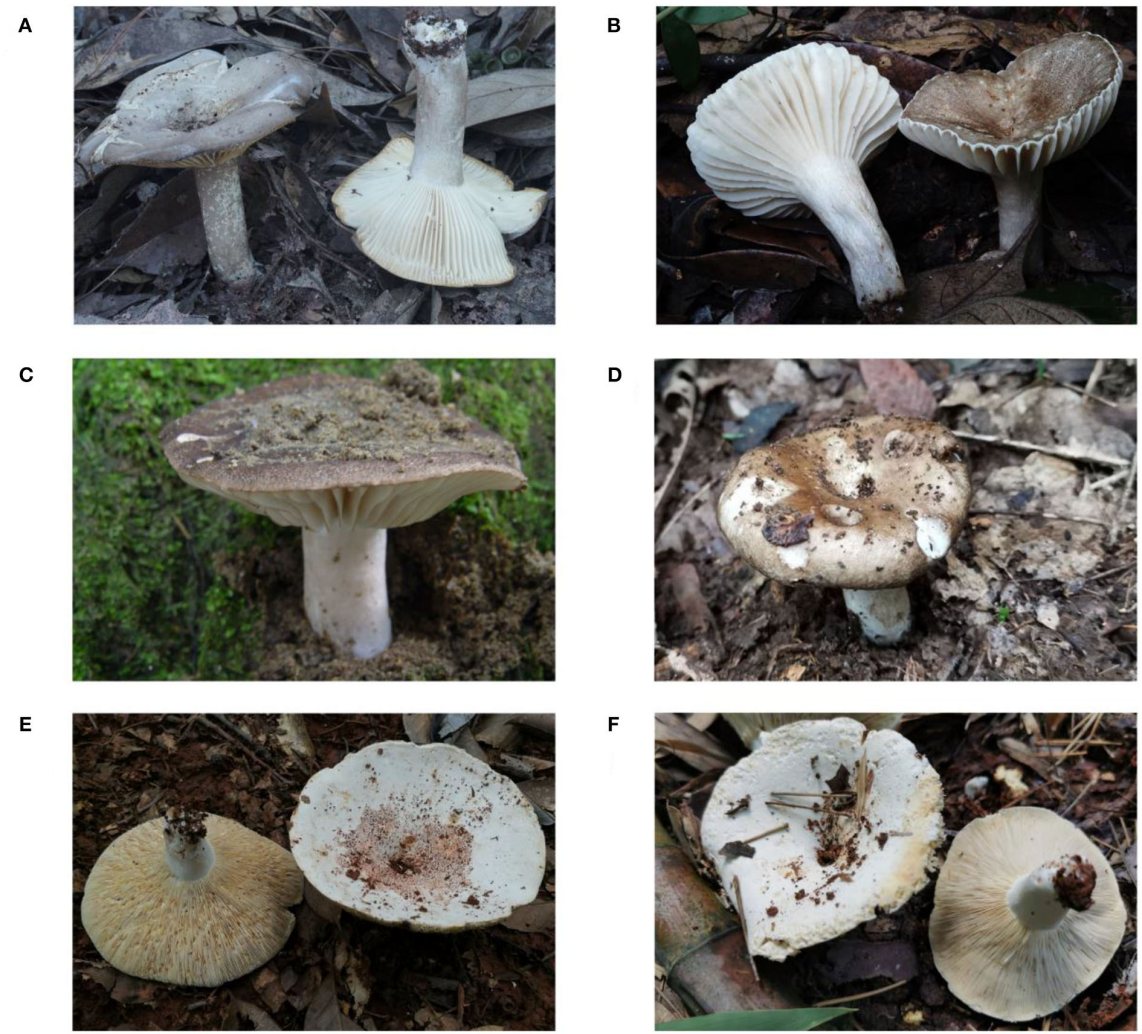
Basidiomata of *Russula* species. **(A)** and **(B)**: *Russula subnigricans*; **(C)**: *R. nigricans*; **(D)**: *R. densifolia*; and **(E)** and **(F)**: *R. japonica*.

Rapid and accurate identification of poisonous mushroom species is of vital importance for the prevention, source investigation, and accurate and effective treatment of mushroom poisoning. At present, poisonous mushroom species are identified mainly through morphological evidence, molecular biology, and toxin analysis. Morphological identification requires professional knowledge of fungal taxonomy. Due to the diversity of species in the *Russula* genus, it is difficult for the public to distinguish poisonous species and edible species because of their similar morphology. The specimens examined for this study indicate that *Russula nigricans* and *Russula densifolia* closely resembled *R. subnigricans*, and *R. japonica* resembles other edible *Russula* species, making species identification difficult (Figure 1). With the development of molecular biology and analytical chemistry, DNA barcoding techniques and high-performance liquid chromatography (HPLC) have been used for species identification and toxin analysis (White, 1990; Enjalbert et al., 1992; Parnmen et al., 2016). However, the limitations of expensive equipment and professional operations are not suitable for primary institutions and rural areas.

In recent years, loop-mediated isothermal amplification (LAMP) has been used in environmental monitoring and detection of pathogenic microorganisms because of its simplicity, rapidity, high specificity, and sensitivity (Wang et al., 2008; Chen et al., 2013; Yang et al., 2018; Baek et al., 2020; Waliullah et al., 2020). More importantly, the results of LAMP reactions with hydroxy naphthol blue (HNB) allow for visual discrimination without the use of costly specialized equipment (Goto et al., 2009; Naraporn and Choopara, 2019). Notomi first developed LAMP as a potential method for nucleic acid amplification, and the reaction is carried out at a constant temperature ranging from 60 to 65°C using a set of four primers catalyzed by a DNA polymerase with strand displacement activity called Bst DNA polymerase (Notomi et al., 2000). A set of primers reflects specificity, and high sensitivity is due to the fact that it generates a vast quantity of amplification products (10^9^-10^10^ times) in a short period of time (Nagamine et al., 2002; Notomi et al., 2015). The reaction time is ~1 h, but the addition of loop primers reduces the consumption time (Nagamine et al., 2002).

LAMP has been used in the detection of lethal mushrooms belonging to *Amanita, R. senecis*, and *C. molybdites* in the field of mushroom identification to date (Vaagt et al., 2013; He et al., 2019a; Wang et al., 2021, 2022). In this study, LAMP, as a rapid and effective method, is applied to detect the deadly poisonous species *R. subnigricans* and the widely distributed toxic species *R. japonica*.

## Materials and methods

### Mushroom samples

Table 1 lists 32 mushroom samples from 18 species that were used in this study. *R. subnigricans* samples were collected from nine different poisoning incidents, and mushrooms from other Russulaceae species were collected in the wild or from the CDC in China. All specimen vouchers, including samples S-81 and S-114, have been deposited in the Mycological Herbarium of Hunan Normal University (MHHNU).

**Table 1:** Mushroom samples used in this study and their related information.

**Species**	**Specimen no**.	**Geographic origin**	**Collection time**	**ITS Genebank** **accession no**.
*Russula subnigricans*	MHHNU 30977	Xiangtanxian, Xiangtan, Hunan	2016	OM760645
*R. subnigricans*	MHHNU 30982	Pingjiangxian, Yueyang, Hunan	2016	OM760659
*R. subnigricans*	MHHNU 31027	Linxiangxian, Yueyang, Hunan	2017	MK430039
*R. subnigricans*	MHHNU 31035	Changshaxian, Changsha Guizhou	2017	OM760671
*R. subnigricans*	MHHNU 31193	Xiangtanxian, Xiangtan, Hunan	2018	OM760673
*R. subnigricans*	MHHNU 31198	CDC of Xiangxiang, Hunan	2018	OM760674
*R. subnigricans*	MHHNU 31579	CDC of Nanping, Fujian	2019	OM760844
*R. subnigricans*	MHHNU 31580	CDC of Zhejiang	2019	OM760843
*R. subnigricans*	MHHNU 8115	Xinshaoxian, Shaoyang, Hunan	201	OM760652
*R. nigricans*	MHHNU 31329	Zixi Mountain, Chuxiong, Yunnan	2018	OM760678
*R. nigricans*	MHHNU 31554	Sangzhixian, Zhangjiajie, Hunan	2019	OM760733
*R. nigricans*	MHHNU 7172	Liuyangshi, Changsha, Hunan	2007	OM760649
*R. nigricans*	MHHNU 7951	Yuelu Mountain, Changsha, Hunan	2014	OM760648
*R. densifolia*	MHHNU 10137	Yongxingxian, Chenzhou, Hunan	2019	OM760656
*R. crustosa*	MHHNU 7960	Yuelu Mountain, Changsha, Hunan	2014	OM760651
*R. crustosa*	MHHNU 31563	Hefengxian, Enshi, Hubei	2019	OM760758
*R. mairei*	MHHNU 31537	Sangzhixian, Zhangjiajie, Hunan	2019	OM760730
*R. pulchra*	MHHNU 31544	Sangzhixian, Zhangjiajie, Hunan	2019	OM760740
*R. chiui*	MHHNU 31570	Hefengxian, Enshi, Hubei	2019	OM760756
*R. risigallina*	MHHNU 31587	Sangzhixian, Zhangjiajie, Hunan	2019	OM760757
*R. virescens*	MHHNU 7504	Liuyangshi, Changsha, Hunan	2011	KU552087
*R. zvarae*	MHHNU 8475	Changsha, Hunan	2015	OM760653
*R. griseocarnosa*	MHHNU 8652	Sangzhixian, Zhangjiajie, Hunan	2015	MK172816.1
*R. senecis*	MHHNU 9055	Youxian, Zhuzhou, Hunan	2017	OM760657
*R. sp*.	MHHNU 8478	Ningxiang, Changsha, Hunan	2015	OM760655
*R. rosea*	S-81	Sangzhixian, Zhangjiajie, Hunan	2019	OM760771
*Lactarius kesiyae*	MHHNU 31638	Hefengxian, Enshi, Hubei	2019	OM760761
*R. japonica*	MHHNU 31484	Ningxiang, Changsha, Hunan	2019	OM760732
*R. japonica*	MHHNU 31881	Ningxiang, Changsha, Hunan	2020	OM760763
*L. vividus*	MHHNU 31821	Changde, Hunan	2019	OM760767
*R. cyanoxantha*	S-114	Sangzhixian, Zhangjiajie, Hunan	2019	OM760772
*R. japonica*	MHHNU 31366	Youxian, Zhuzhou, Hunan	2018	OM760677

### DNA extraction, polymerase chain reaction amplification, and internal transcribed spacer sequencing

Approximately 10 mg of each dried sample was weighed. Total genomic DNA was extracted with the Fungal DNA Mini kit (Omega, USA). Internal transcribed spacers (ITSs) 4 and 5 were used to amplify ITS sequences. Each polymerase chain reaction (PCR) mixture contained 1 × PCR buffer, 1.5 mM MgCl_2_, 0.2 mM dNTPs, 0.4 μM of each primer, 1.25 U of Taq polymerase, and 1 μl DNA template in a total volume of 25 μl. PCRs were run on an Eppendorf Mastercycler thermal cycler (Eppendorf, Inc., Germany) as follows: initial denaturation at 94°C for 4 min; followed by 30 cycles of 94°C for 30 s, 54°C for 30 s, and 72°C for 30 s; and a final extension at 72°C for 8 min. Amplified PCR products were detected using gel electrophoresis on 1% agarose gels and then sequenced at Tsingke Biological Technology (China).

### Design of primers for LAMP and PCR

**Figure 2:**
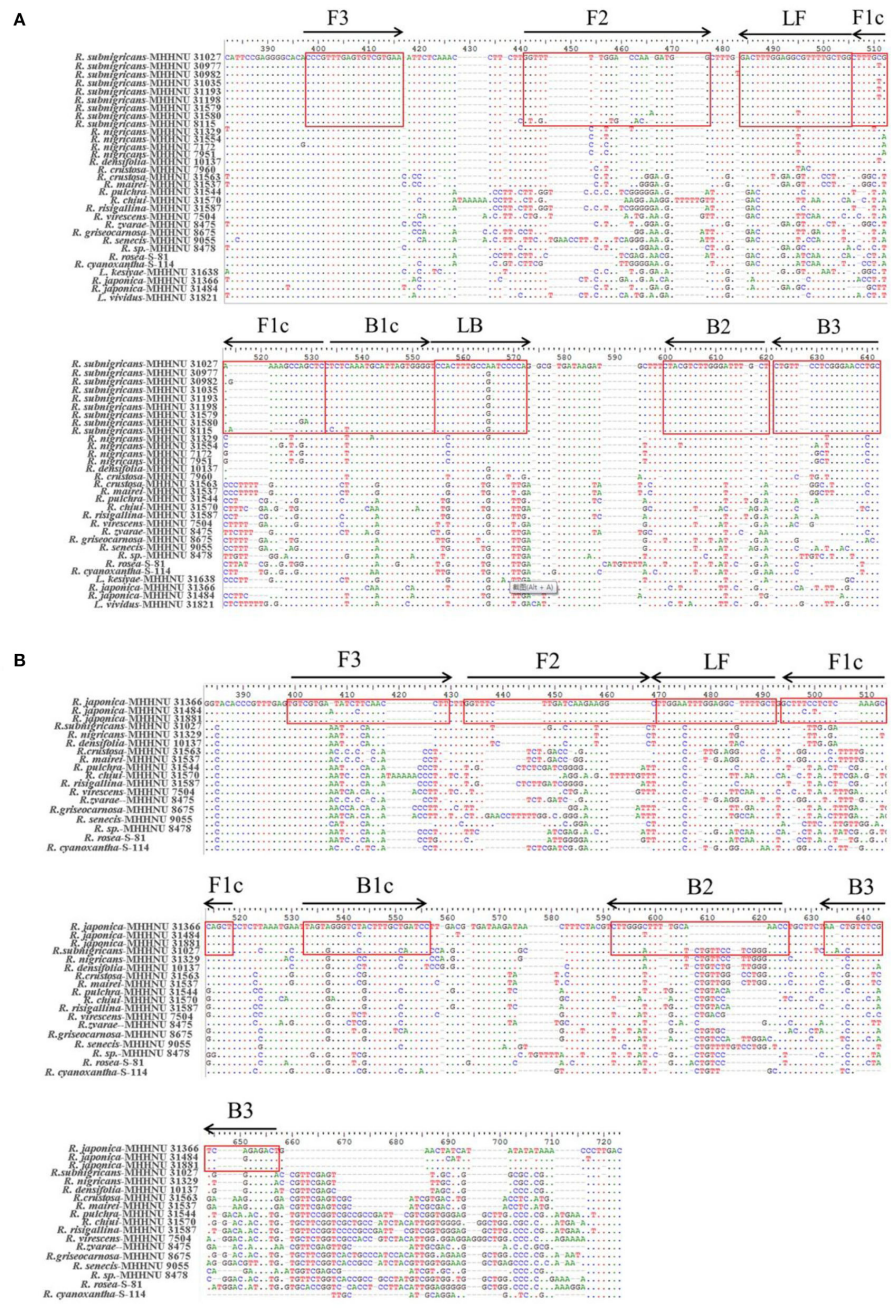
Multiple-sequence alignment of internal transcribed spacer (ITS) of *Russula* samples. The target regions used for designing loop-mediated isothermal amplification (LAMP) primers were labeled. LAMP primers for *R. subnigricans* **(A)** and LAMP primers for *R. japonica* **(B)**.

A set of six specific primers based on the published ITS sequences of *R. subnigricans* and a set of five specific primers (GenBank accession Nos. MK430039 and OM760677) based on the sequences of *R. japonica* were designed using the professional online software PrimerExplorer V5 (http://primerexplorer.jp/lampv5e/index.html). In the case of *R. subnigricans*, a forward inner primer (FIP) consisting of the complementary sequences of F1c and F2, a backward inner primer (BIP) consisting of B1c and B2, and two outer primers (F3 and B3), and two loop primers (LF and LB) were used for LAMP. For *R. japonica*, only one loop primer was suitable for *R. japonica*. Multiple-sequence alignment of ITS of *Russula* samples and the target regions used to design LAMP primers are labeled and seen in Figure 2. Species-specific PCR primers for *R. subnigricans* were designed using Primer 5.0 software, including Rs-f (forward primer of *R. subnigricans*) and Rs-r (reverse primer of *R. subnigricans*). The primers (PAGE) were synthesized by Tsingke Biological Technology (China), and their sequences and lengths are presented in Table 2.

**Table 2:** Information on the species-specific primers used for loop-mediated isothermal amplification (LAMP).

**Primer name**	**Primer type**	**Primer sequence (5^′^ → 3^′^)**	**Primer length (bp)**
Rs-primers	F3	CCCGTTTGAGTGTCGTGAA	19
	B3	CAGGTTCCCGAGGAACAG	18
	FIP	GAGCTGGCTTTTCGCAAGGCTAATGGTTTTTGGACCAAGATGGC	44
	BIP	CTCTCAAATGCATTAGTGGGGTATAAGCAAATCCCAAGACGTAGA	46
	LF	CAGCAAAACACCTCCAAAGTCC	22
	LB	CCACTTTGCCGATCCCCA	18
Rj-primers	F3	TGTCGTGATATCTTCAACCTT	22
	B3	AGTCTCTGACGAGACAGTT	19
	FIP	AGCTGGCTTTGAGAGGAAAGCTAATGGTTTCTTGATCAAGAAGGC	45
	BIP	TAGTAGGGTCTACTTTGCTGATCCTATAGGTTTGCAAAAGCCCAAG	46
	LF	AGCAAAAGCCTCCAAATTCCAA	22
PCR primers	Rs-f	GCTTTGGACTTTGGAGGCG	19
	Rs-r	ACAGAGCAAATCCCAAGAC	19

Rs represents Russula subnigricans; Rj represents R. japonica.

### Optimization of LAMP reaction temperatures

Real-time quantitative LAMP (RT-qLAMP) was carried out in a 10-μl reaction mixture: 1× ThermoPol buffer (20 mM Tris-HCl, 10 mM (NH_4_)_2_SO_4_, 10 mM KCl, 2 mM MgSO_4_, and 0.1% Triton X-100, pH 8.8), 4 mM MgSO_4_, 1.4 mM dNTP mix, 1.28 μM FIP, 1.28 μM BIP, 0.12 μM F3, 0.12 μM B3, 0.64 μM LF, 0.64 μM LB (absent in the LAMP reaction of *R. japonica*), 3.2 U of Bst DNA polymerase (NEB, USA), 5 ng DNA template, 150 μM 0.5 × SYBR Green I, and ddH_2_O to a total volume of 10 μl. These reactions were performed using the QuantStudio 5 Real-Time PCR System (ABI, USA). The reaction mixtures were incubated at different temperatures ranging from 60 to 65°C for 40 min. A real-time fluorescence detection system was used for monitoring the LAMP reactions. As each cycle was set to 1 min, the threshold time (*Tt*) value was equivalent to the cycle time (*Ct*). The SYBR Green I data were plotted as the relative fluorescence signal vs. time, with each cycle set to 1 min at a constant temperature of 62°C.

### LAMP reaction and product detection

**Figure 3:**
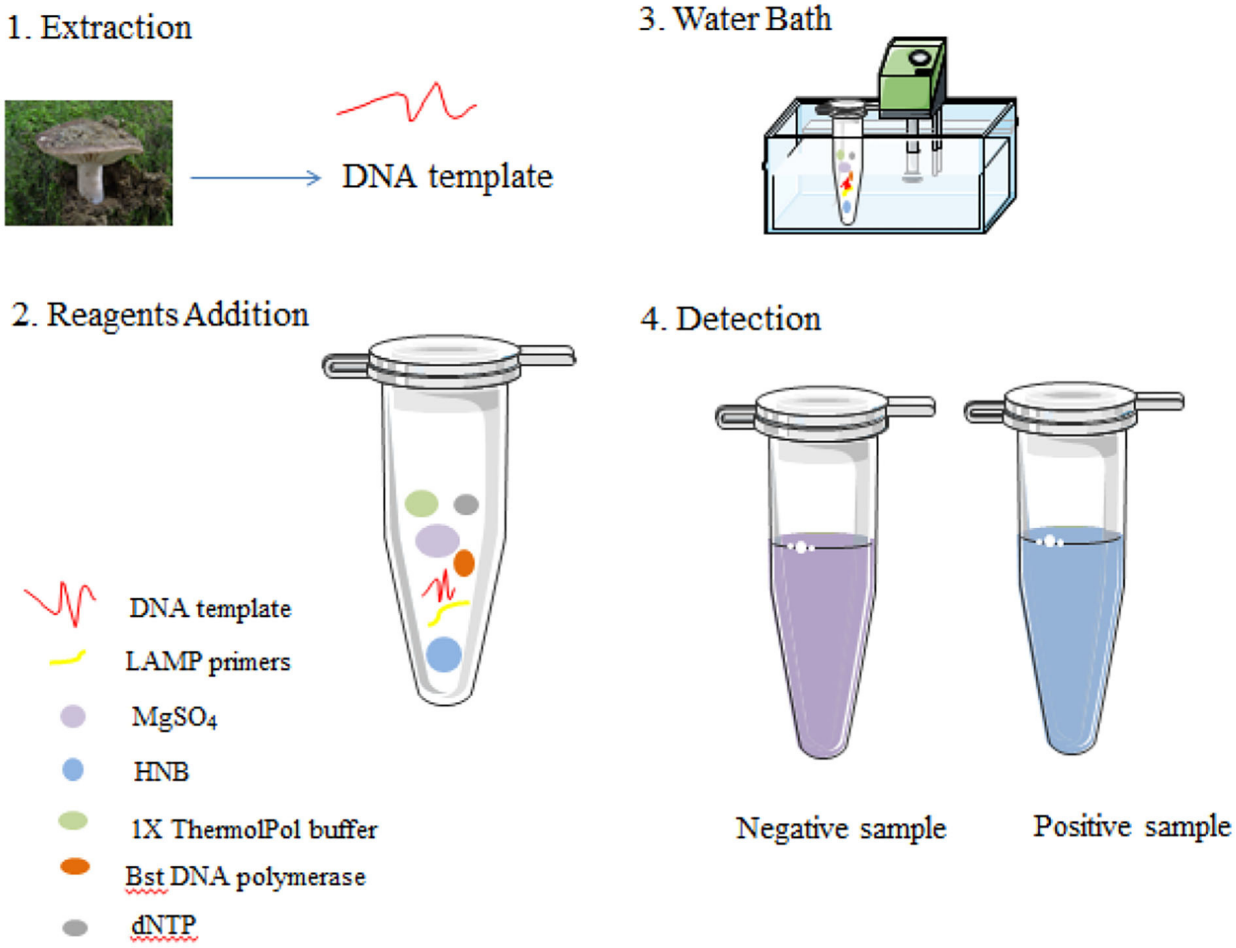
Schematic illustration of the LAMP assay.

**Figure 4:**
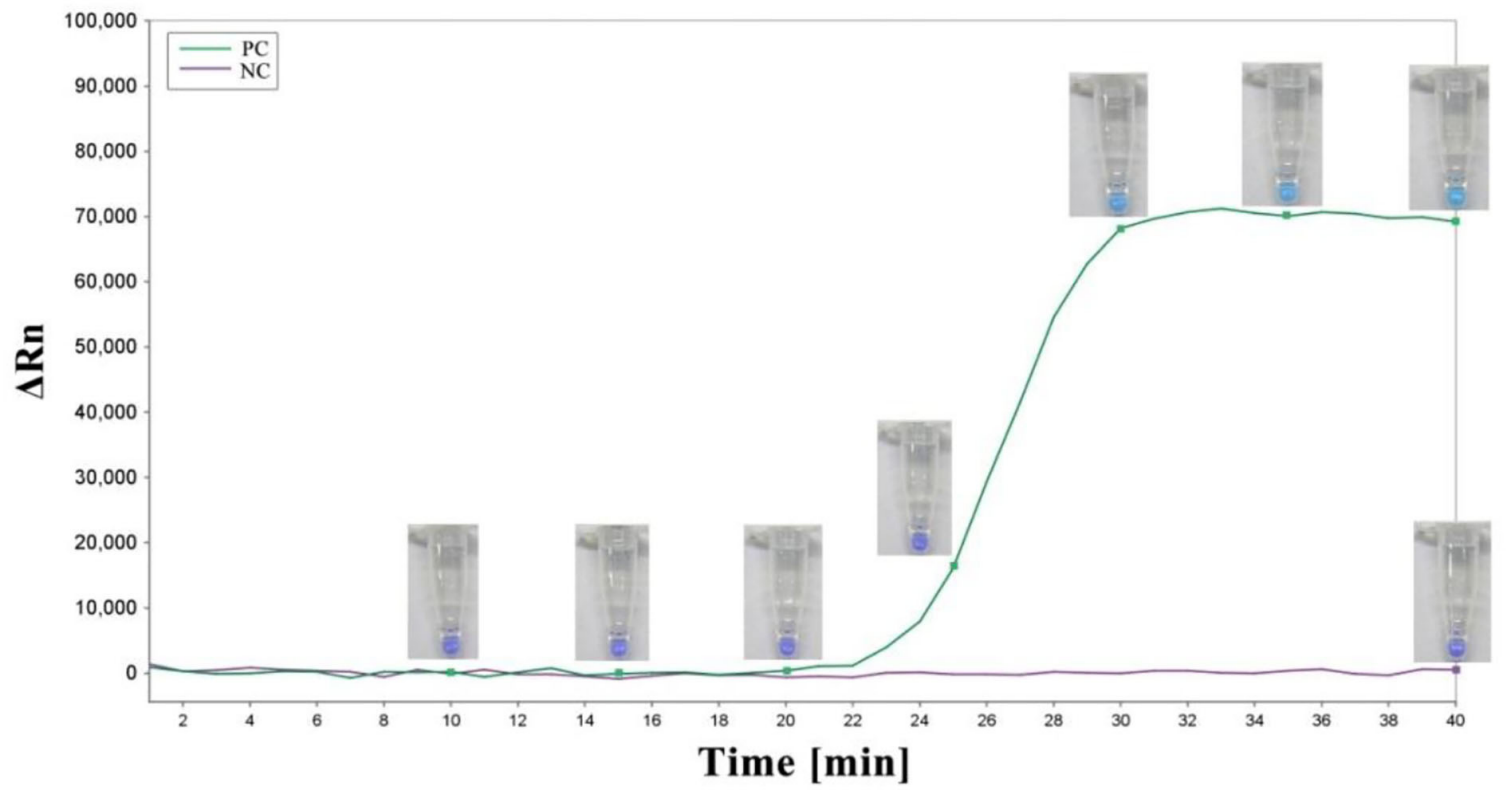
The amplification plot and corresponding colors of the LAMP mixture over time. A sky blue color was observed in the case of amplification, whereas the negative control remained violet after the reaction. PC, positive control; NC, negative control.

As previously described, the LAMP reaction was carried out in a 10-μl reaction mixture with HNB replacing 0.5 × SYBR Green I. Each reaction was performed in a 0.2-ml tube with a water bath incubated at 62°C for 45 min and finally at 80°C for 10 min to terminate the reaction (Figure 3). Conventional agarose gel electrophoresis was used to analyze DNA amplification products. In addition, in our study, another low-pollution approach,direct visual inspection of LAMP mixtures with HNB by the naked eye, was also used to detect amplification products of LAMP. Real-time measurement of SYBR Green I-based fluorescence was used to verify the HNB staining method (Figure 4).

### Tests of specificity and sensitivity of the LAMP assay and PCR

To test the specificity of the LAMP primers for *R. subnigricans*, genomic DNA from 31 wild samples, including *R. subnigricans* collected from nine different poisoning incidents, was used in 45-min LAMP reactions. To test the specificity of LAMP and PCR, specific PCR primers were designed to amplify the genomic DNA of *R. subnigricans*. For the specificity of the LAMP primers of *R. japonica*, genomic DNA of 19 wild samples, including *R. japonica* collected from three wild points, was used for 45-min LAMP reactions. To determine the detection limit, LAMP and PCR assays were performed using a series of 10-fold dilutions of genomic DNA from *R. subnigricans* (5 ng to 50 fg) at 45 min intervals.

## Results

### Optimized temperature of the LAMP reaction

To optimize the temperature, LAMP reactions were carried out at different temperatures from 60 to 65°C at 1°C intervals (each *Tt* value displayed is the mean of three replications). The threshold time (*Tt*) values of different temperatures obtained from the RT-qPCR system were analyzed, and the temperature of 62°C was found to be optimal in terms of the lowest *Tt* (Table 3).

**Table 3:** Threshold times (*Tt*) of LAMP at different temperatures.

**Temperature (**°**C)**	**60**	**61**	**62**	**63**	**64**	**65**
**Tt value (min)**	25.037	23.551	21.187	24.414	25.744	28.512

Each displayed Tt value is the mean of three replications.

### Specificity of LAMP and PCR

**Figure 5:**
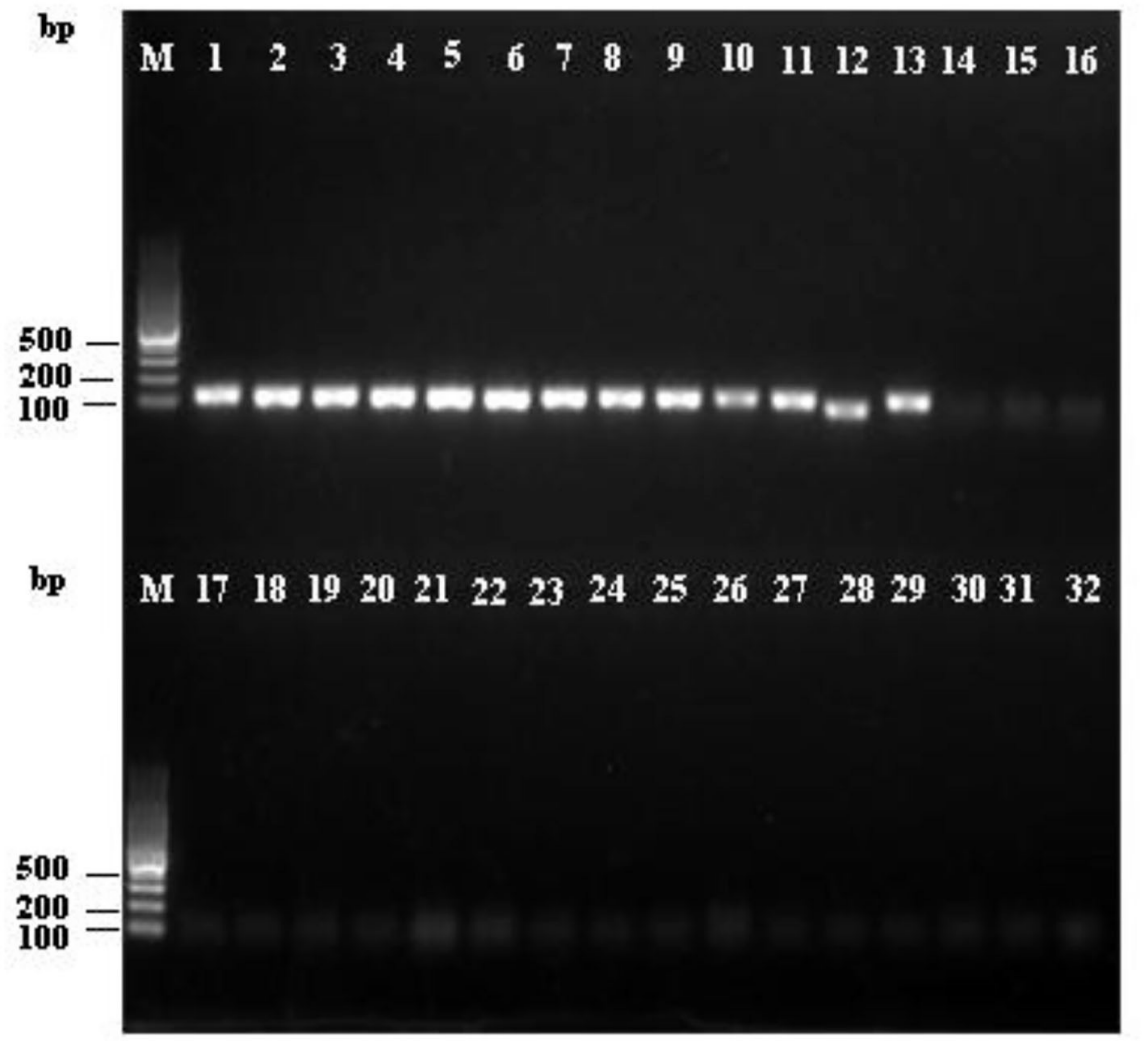
Specificity of the polymerase chain reaction (PCR) assay for *R. subnigricans*. Amplification was detected by agarose gel electrophoresis of PCR amplification products. M: 100 bp ladder; lanes 1–9: *R. subnigricans*; lanes 10–13: *R. nigricans*; lane 14: *R. densifolia*; lanes 15–16: *R. crustosa*; lane 17: *R. mairei*; lane 18: *R. pulchra*; lane 19: *R. chiui*; lane 20: *R. risigallina*; lane 21: *R. virescens*; lane 22: *R. zvarae*; lane 23: *R. griseocarnosa*; lane 24: *R. senecis*; lane 25: *R*. sp.; lane 26: *R. rosea*; lane 27: *Lactarius kesiyae*; lane 28: *R. japonica*; lane 29: *R. japonica*; lane 30: *Lactarius vividus*; lane 31: *R. cyanoxantha*; and lane 32: ddH_2_O (blank control).

**Figure 6:**
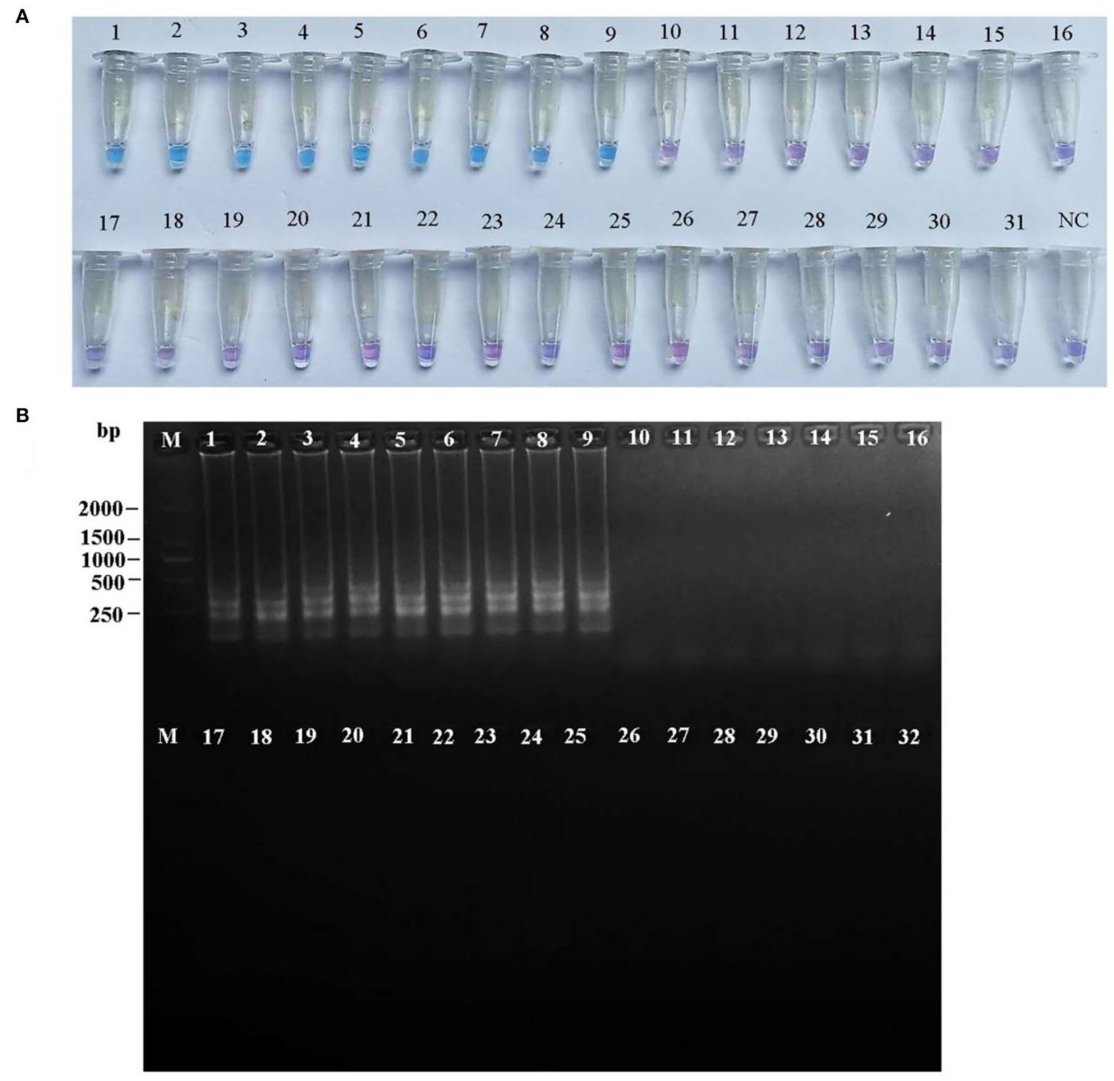
Specificity of the LAMP assay for *R. subnigricans*. Hydroxy naphthol blue (HNB) dye staining of LAMP products **(A)** and agarose gel electrophoresis of LAMP products **(B)**. M: 100 bp ladder; lanes 1–9: *R. subnigricans*; lanes 10–13: *R. nigricans*; lane 14: *R. densifolia*; lanes 15–16: *R. crustosa*; lane 17: *R. mairei*; lane 18: *R. pulchra*; lane 19: *R. chiui*; lane 20: *R. risigallina*; lane 21: *R. virescens*; lane 22: *R. zvarae*; lane 23: *R. griseocarnosa*; lane 24: *R. senecis*; lane 25: *R*. sp.; lane 26: *R. rosea*; lane 27: *L. kesiyae*; lane 28: *R. japonica*; lane 29: *R. japonica*; lane 30: *L. vividus*; lane 31: *R. cyanoxantha*; and lane 32: ddH_2_O (blank control).

To confirm the specificity of the LAMP and PCR primers for *R. subnigricans*, a total of 31 samples of mushroom species in Russulaceae, including nine *R. subnigricans* specimens from different poisoning incidents, 15 other *Russula* species, and two *Lactarius* species, were used to test the specificity of the LAMP assay. As seen in Figure 5, the amplification products of the PCR reactions were analyzed using agarose gel electrophoresis, while the LAMP reactions were analyzed using agarose gel electrophoresis and HNB dye staining (Figure 6). Not only *R. subnigricans* but also *R. nigricans* were successfully amplified by PCR, and bright bands were observed. The two LAMP detection methods produced consistent results. Positive reactions were observed in all *R. subnigricans* samples from different poisoning incidents, with typical ladder-like bands and sky blue mixtures, whereas no bands were observed for other *Russula* and *Lactarius* samples, and the color in the tubes remained violet after the reaction.

**Figure 7:**
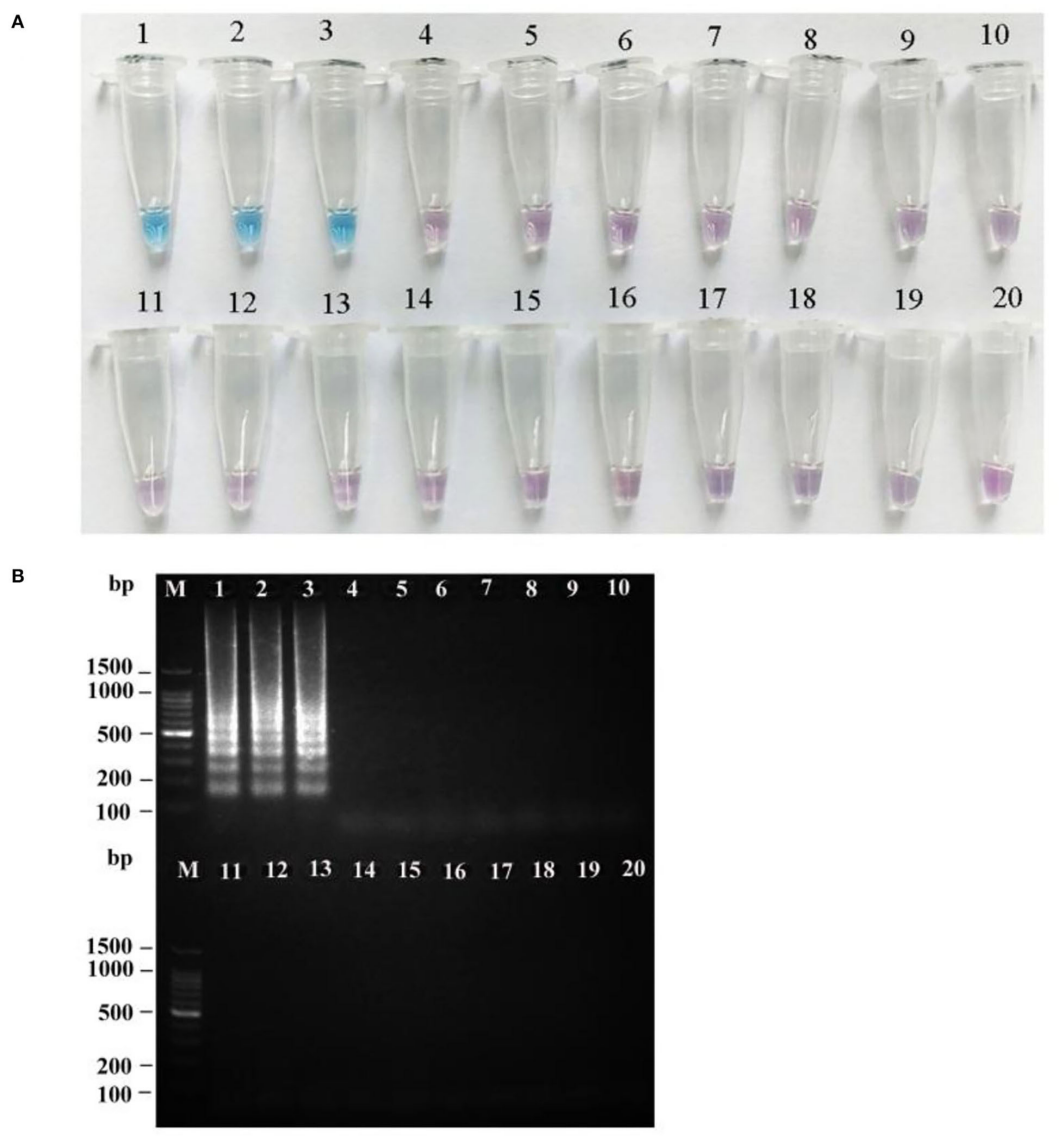
Specificity of the LAMP assay for *R. japonica*. HNB dye staining of LAMP products **(A)** and agarose gel electrophoresis of LAMP products **(B)**. M: 100 bp ladder; lanes 1–3: *R. japonica*; lane 4: *R. subnigricans*; lane 5: *R. nigricans*; lane 6*: R. densifolia*; lane 7: *R. crustosa*; lane 8: *R. crustosa*; lane 9: *R. mairei*; lane 10: *R. pulchra*; lane 11: *R. chiui*; lane 12: *R. risigallina*; lane 13: *R. virescens*; lane 14: *R. zvarae*; lane 15: *R. griseocarnosa*; lane 16: *R. senecis*; lane 17: *R*. sp.; lane 18: *R. rosea*; lane 19: *R. cyanoxantha*; and lane 20: ddH_2_O (blank control).

To verify the specificity of the LAMP primers for *R. japonica*, a total of 19 samples of mushroom species Russulaceae, including three *R. japonica* specimens from different locations in the wild and 15 other *Russula* species, were used to test the specificity of the LAMP assay. As seen in Figure 7, the results of the two methods of LAMP-positive detection of *R. japonica* were consistent with those of *R. subnigricans*. Three *R. japonica* were found with typical ladder-like bands and sky blue mixtures from all *R. japonica* samples, contrary to the patterns from other *Russula* samples with no bands, and with violet mixtures in the tubes after amplification.

### Sensitivity of LAMP and PCR

**Figure 8:**
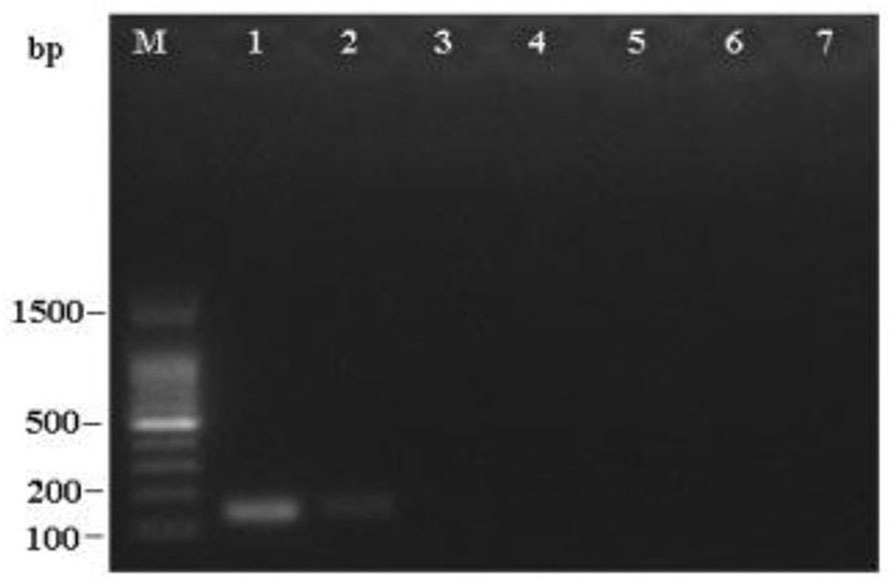
Sensitivity of the PCR assay for *R. subnigricans*. A dilution series of *R. subnigricans* DNA was prepared as follows: (1) 5 ng; (2) 500 pg; (3) 50 pg; (4) 5 pg; (5) 500 fg; (6) 50 fg; and (7) no template for negative control.

**Figure 9:**
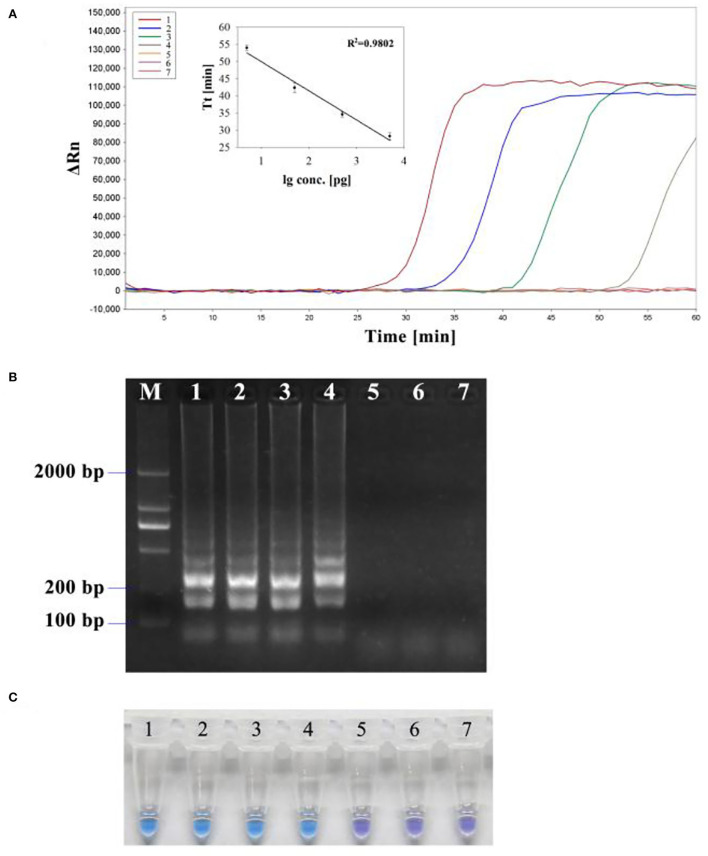
Sensitivity of the LAMP assay for *R. subnigricans*. A dilution series of *R. subnigricans* DNA was prepared as follows: (1) 5 ng; (2), 500 pg; (3) 50 pg; (4) 5 pg; (5) 500 fg; (6) 50 fg; and (7) no template for negative control. **(A)** Real-time quantitative LAMP (RT-qLAMP) analysis with different DNA concentrations. The standard curve based on this dilution series showed a linear relationship between the log of the quantity of initial template DNA (lg conc.) and threshold time (*Tt*). The coefficient of determination (*R*^2^) of the linear regression was 0.9802. **(B)** Electrophoresis-LAMP with different DNA concentrations. **(C)** HNB-LAMP with different DNA concentrations.

To determine the detection limit, LAMP and PCR reactions were performed on serial 5 ng to 50 fg 10-fold diluted DNA templates of *R. subnigricans*. The detection of PCR is 500 pg/reaction (Figure 8). A real-time fluorescent quantitative system, gel electrophoresis, and HNB dye were used to detect the amplification products of LAMP. LAMP detection limits of the three methods for *R. subnigricans* analysis were identical and 100-fold higher than PCR: 5 pg per reaction (Figure 8). These results suggest that the detection sensitivity of the LAMP mixture with HNB dye (HNB-LAMP) for *R. subnigricans* is comparable to that of electrophoresis-LAMP and RT-qLAMP (Figure 9).

## Discussion

Fungi of the genus *Russula*, widely distributed worldwide, play an important role in all ecosystems and the natural kingdom (Buyck et al., 2018; Adamčík et al., 2019). *R. subnigricans* is easily confused morphologically with edible wild mushrooms such as *R. nigricans* and *R. densifolia*, which is consistent with their phylogenetic relationships, since all are members of the *Russula* subsection *Nigricantinae* (Wang et al., 2020). *R. japonica* is a poisonous mushroom most difficult to differentiate from edible species because the subgenus to which *R. japonica* belongs also includes many famous edible species, such as *R. delica* and *Russula brevipes* (Wang et al., 2020). The lethality of *R. subnigricans* and the universality of *R. japonica* cause poisoning events to occur frequently from May to September of every year, especially in southern China (Matsuura et al., 2009; Lin et al., 2015; Cho and Han, 2016; Li et al., 2020, 2021, 2022).

Loop-mediated isothermal amplification, with the qualities of high sensitivity and specificity, offers a novel, simple, and rapid poisonous mushroom detection approach based on DNA amplification, which eliminates the stages of DNA denaturation and thermal cycling. Vaagt et al. (2013) first used LAMP in the detection of the poisonous mushroom *Amanita phalloides*. He et al. (2019a) achieved the detection of lethal *Amanita* species and introclade discrimination of lethal *Amanita* species with LAMP, while hyperbranched rolling circle amplification (HRCA) could discriminate it as introclade or intraclade species. However, HRCA requires an extra ligation of the padlock probe (1 h) before isothermal amplification, which is more time-consuming than LAMP though it has high specificity (He et al., 2019a,b). In the detection of *Mycoplasma ovipneumoniae*, the reaction times of real-time recombinase polymerase amplification (RT-RPA) and lateral flow strip recombinase polymerase amplification (LFS-RPA) are shorter than LAMP *via* a lateral flow dipstick (LAMP-LFD) assay, but the effectiveness of RT-RPA and LFS-RPA in the detection needs to be further validated (Zhang et al., 2019; Wang et al., 2020). Furthermore, LAMP primers for detecting poisonous mushrooms all are based on ITS regions, and according to various families and genera, we can design specific primers based on other gene fragments, such as nrLSU, *rpb1*, and *rpb2* (Vaagt et al., 2013; He et al., 2019a; Wang et al., 2021, 2022).

In this study, two sets of species-specific LAMP primers based on ITS and to specifically detect *R. subnigricans* and *R. japonica* successfully were matched as expected. Although *R. subnigricans, R. nigricans*, and *R. densifolia* have close phylogenetic relationships, LAMP could specifically discriminate *R. subnigricans* from other *Russula* species, while species-specific PCR primers failed to differentiate *R. subnigricans* from *R. nigricans*. *R. japonica* was also specifically detected using LAMP (Table 4). The results indicated that the LAMP assay could reach the target detection compared with traditional PCR though LAMP is less specific than HRCA (He et al., 2019a). Furthermore, in the sensitivity test, the results demonstrated that the detection limits of visual HNB-LAMP, electrophoresis-LAMP, and RT-qLAMP assays could reach 0.5 pg/μl, indicating that the detection sensitivity was 100-fold higher than that of PCR (Table 4). Compared with the detection of other species using LAMP, the detection limit was half of that in *Amanita* and was higher than that in *R. senecis* and *C. molybdites* (He et al., 2019a; Wang et al., 2021, 2022). In addition, LAMP showed excellent advantages of strong specificity and high sensitivity, which have also been observed in other fields, such as *Cucurbit Leaf Crumple Virus*, Avian infectious laryngotracheitis (AILT), and SARS-CoV-2 (Ghodrati et al., 2017; Waliullah et al., 2020; Yan et al., 2020; Yu et al., 2021). More importantly, in terms of simplicity, the HNB-LAMP visual system was validated using agarose gel electrophoresis, and the results of HNB-LAMP assays were consistent with those of agarose gel electrophoresis. With the addition of HNB as a visual indication, the result is easy to observe with the naked eye without opening the tube and causing aerosol pollution (Goto et al., 2009; Naraporn and Choopara, 2019). Owing to the strand displacement activity of DNA polymerase superseding thermal denaturation steps and the addition of loop primers, the result can be obtained in 45 min, much less than the reaction time of other isothermal amplification technologies like helicase-dependent amplification (HAD), strand displacement amplification (SDA), and HRCA (Nagamine et al., 2002; Barreda-García et al., 2018; He et al., 2019a,b; Kolm et al., 2019; Hu et al., 2020; Chi et al., 2022). Moreover, the temperature of LAMP assays ranging from 60 to 65°C confirmed their feasibility for field applications. By eliminating the need for expensive specific facilities and reagents, LAMP-based assays coupled with HNB dye will be more suitable for primary institutions and remote areas (Tomita et al., 2008; Pandey et al., 2018).

**Table 4:** Specificity and sensitivity detection results of *R. subnigricans*.

**Detection method**	**Specificity (positive species)**	**Sensitivity (pg)**
LAMP	*R. subnigricans*	5
PCR	*R. subnigricans*; *R. nigricans*	500

In conclusion, LAMP-based assays examined in this study proved to be a promising, well-performing, rapid, highly specific, and sensitive detection method and to be suitable for on-site applications because of their feasibility and cost-effectiveness in terms of money and time. Furthermore, LAMP-based assays are expected to have broad application prospects in other potential fields.

## Data availability statement

The datasets presented in this study can be found in online repositories. The names of the repository/repositories and accession number(s) can be found in the article/supplementary material.

## Author contributions

ZC and PL conceived and designed the experiments, and wrote this manuscript. PL, ZH, and ZJ carried out the LAMP assay. All authors contributed to the article and approved the submitted version.

## Funding

This study was supported by the Key Research and Development Program of Hunan province (Grant No. 2020SK2103) and the Biodiversity Survey and Assessment Project of the Ministry of Ecology and Environment, China (Grant No. 2019HJ2096001006).

## Conflict of interest

The authors declare that the research was conducted in the absence of any commercial or financial relationships that could be construed as a potential conflict of interest.

## Publisher's note

All claims expressed in this article are solely those of the authors and do not necessarily represent those of their affiliated organizations, or those of the publisher, the editors and the reviewers. Any product that may be evaluated in this article, or claim that may be made by its manufacturer, is not guaranteed or endorsed by the publisher.
